# Two Cases of Pulmonary Hæmorrhage, Speedily and Successfully Cured by Abstinence from Liquids

**Published:** 1792

**Authors:** William Davidson

**Affiliations:** Apothecary in London.


X. *Two Cafes of pulmonary Hæmorrhage, fpeedily and fuccefsfully cured by Abftinence from Liquids. By the Same.*

ON the 6th day of March, 1792, I was requefted to vifit Mr. S——, a man of a florid complexion, full habit of body, and about forty-five years of age. He had been much affected with head-ach, and hard dry cough, for three or four weeks. His pulfe was now full, frequent, frequent, hard, and quick; and the veins upon his hands and arms were ſo much diſtended, that they appeared as if ready to burſt. The cough was almoſt inceſſant, attended with conſiderable expectoration of florid frothy blood, which made its appearance this morning, after a ſevere fit of coughing, and his head-ach ſtill continued.

The plan I followed was the following:

I took, from a large orifice in the arm, twelve ounces of blood, which from the long time it remained fluid after being taken from the arm, and the conſequent appearance of (what is commonly called) inflammation, both indicating the great action of the ſanguiferous ſyſtem, ſeemed to point out the neceſſity of employing the moſt vigorous antiphlogiſtic treatment. Much danger was alſo to be apprehended from the enlargement of the opening of the ruptured veſſel.

Accordingly, I ordered him a ſaline draught, with antimonial wine, to be taken every three hours, adding to the night draught ſome ſyrup of white poppies, and an opening ſaline draught to be taken the morning following, and repeated every other morning.

This courſe of medicine, together with abſtinence from animal food, and a ſtrict adherence to a light cooling diet, was regularly purſued for

 three three days; during which time the bleeding, although moderated, ftill continued, but the cough was much better.

9th. He was directed to continue the fame diet, and to avoid much exercife; and the turgid ftate of the veins of his hands admonifhing me that his veffels were ftill too full, it occurred to me to advife, inftead of a fecond bleeding, that he fhould drink as fparingly as poffible; from which I thought the veffels would become lefs full, and the ruptured veffel have a greater chance of uniting than when conftantly diftended by drinking; and that, if I could avoid taking away more blood, my patient would recover from his indifpofition much fooner than if I refted the chief ftrefs of the cure upon this operation. He was, therefore, allowed only a pint of liquid, including tea and every other kind of drink, (all of which were given cold) in the twenty-four hours. When thirfty, I recommended it to him to fuck an orange or lemon, inftead of drinking. On former occafions of this kind, viz. in active hæmorrhages, I have prefcribed (as is the common practice) cooling emulfions, milk whey and other diluents, in confiderable quantities, with a view of relaxing the vafcular fyftem, and thereby leffening its increafed creaſed action, not conſidering that the ſtimulus of diſtention kept up this action, and was, therefore, one of the chief things I had to guard againſt. But as there is now little to be dreaded from the Boerhaavian lentor, ſo there is no particular occaſion for the great dilution commonly practiſed, and which ſeems to have been founded upon this doctrine. The medicines preſcribed this day were ſimilar to the former.

10th. I found him very cool, and without cough or expectoration of any kind. The pulſe was ſofter, leſs frequent, and in every reſpect better. The appearance of the cutaneous veins alſo was ſo different, that I was convinced this great alteration for the better was chiefly to be attributed to his having avoided much drinking during the preceding day and night. The draughts kept the body regularly open once or twice a day, and induced a ſoft ſkin and comfortable ſleep. They were, therefore, continued for three days, four every day; and three days more, two every day, ſtill obſerving the ſame rule as to drinking. They always produced the ſame ſalutary effects. From this time the patient was perfectly well, and has remained ſo ever ſince. In this caſe it would appear probable that no particular pneumonic affection exiſted, ed, excepting the bleeding, which was moft probably occafioned by a plethoric ftate of the conftitution and particular determination to the lungs by the cough.

### CASE II.

Soon after my attendance on the above patient, another cafe of hæmoptyfis occurred, but which differed from the former in being attended with confiderable pneumonic affection befides the hæmorrhage. The patient was a tall, thin man, about thirty years of age, of a pale complexion, narrow cheft, and high fhoulders, and had been affected with a fevere cough for nearly four months previoufly to his application to me, accompanied with much yellow expectoration, and was fuppofed by his friends to be in a deep decline. He had no night fweats; but for the laft three weeks had been affected with a continual pain of the right fide; which, as far as I could difcover, did not originate from any rheumatic affection of the external mufcles, but from fome internal difeafe of the thorax, and which I conceived to be a flow inflammation of the lungs, from which, and the violence of the cough, the hæmorrhage proceeded. He applied applied to me in the beginning of April, when he was coughing violently, and bringing up blood in mouthfuls. He had confiderable fever, with a full, hard pulfe. I took from him ten ounces of blood, and prefcribed in every refpect as in the foregoing cafe, enjoining to him great attention not to drink more than a pint of liquid in twenty-four hours; this, and every other rule directed, he regularly obferved for about three weeks, when the bleeding had ceafed for three or four days, and alfo the pain in the fide. But returning imprudently to his former diet, and drinking the ufual quantity as when in health, previoufly, as I fuppofe, to the obftruction or inflammation of the lungs being removed, his cough returned, with fome little appearance of bloody expectoration, mixed with that kind of yellow mucus, which is commonly difcharged by mucous fecreting furfaces when inflamed. Thefe fymptoms, however, were entirely removed in the courfe of ten days, by a fteady attention to the fpare diet, and abftinence from liquids, formerly recommended, and the medicines before ufed.

Since then he has been, and now is, in the moft perfect health, without cough, pain in the fide, or any other thoracic or pneumonic affection.

tion. It occurs to me that this ſecond attack, and the ſucceſs of the ſubſequent treatment, point out the delicate ſituation of the lungs, and alſo the efficacy of this method of cure.

Having related the above two caſes with every neceſſary preciſion, I ſhall beg leave to offer ſome few obſervations on active hæmorrhagy in general, and on that of the lungs in particular. In all active hæmorrhages a plethoric ſtate of the ſyſtem generally exiſts: all the blood veſſels of the body are full, diſtended, and acting vigorouſly; and hence, very commonly, rupture and conſequent hæmorrhage.

Therefore the chief proximate cauſe ſeems to be diſtention and conſequent increaſed action of the veſſels: Dr. Cullen, indeed, adds congeſtion of blood, which certainly may happen either from accidental determination of blood to a part, or ſome particular fault in the original conformation, or acquired relaxation, of the coats of the veſſels of certain parts. But it is well known that hæmorrhages may ariſe from general diſtention, without any particular congeſtion; and, in this caſe, will happen wherever the vaſcular ſyſtem is weakeſt or leaſt ſupported. The proximate cauſe being clearly aſcertained, the the method of cure will appear obvious. Remove the preternatural diſtention of the veſſels, and their action will ſoon diminiſh; then nature, with very little aſſiſtance, will do the reſt. Although this is evidently the caſe, it appears ſingular that, hitherto, almoſt all practitioners have neglected the moſt effectual method of accompliſhing this deſirable purpoſe, viz by abſtinence from liquids. In Dr. Moffat's tranſlation of Aretæus, page 347, are the following words: "The drink ought to be very ſparingly exhibited, for moiſture is diſadvantageous in a dry "diet *." But, although this was written when treating of hæmorrhage, the intentions of Aretæus were only that the aſtringency (upon which he ſeemed to place his chief hope) of his diet might not be weakened by drinking.

The idea of moderate drinking is adopted by Dr. Rowley, in his treatiſe on "Female nervous diſeaſes," publiſhed in 1788. When treating of the "Immoderate flow of the menſes," page 32, he obſerves, "as hæmorrhages ſeldom happen, unleſs there be a ſufficient quantity of blood in the body to rupture the veſ-

* This is an accurate interpretation of the paſſage in queſtion. The words of Aretæus are, ποτὸν δὲ τὸ ξύμπαν ὀλίγον ἔϛω. ξηρῆ γὰρ διαίτῃ ὑγρὸν ἀξύμφορον.

"ſels,

" ſels, one principal part of the cure conſiſts in

" not only obtaining, but preſerving a diminiſhed

" quantity of blood, by a great *abſtinence* from

" liquids; for by this means, the very ſources

" of ſupply are cut off. If little be drank, the

" blood veſſels which are, or have been, diſten-

" ded beyond their proper dimenſions, will gra-

" dually contract themſelves to their original

" ſize, acquire ſtrength daily, and not having

" ſo large a column of blood to circulate, they

" will reſiſt the morbid diſpoſition of nature to

" evacuate ſo violently the catamenia." But, the late celebrated Dr. Cullen, when treating on hæmoptyſis, particularly recommends, that

" every part of the antiphlogiſtic regimen be

" ſtrictly enjoined *," which includes " taking

" in large quantities of mild antiſeptic liquors†:" and ſays, that the phlogiſtic diatheſis is to be taken off by bleeding, more or leſs, according to circumſtances. If, however, the ſtimulus of diſtention is kept up by filling the veſſels with liquids, the good effects of the bleeding are counteracted, and a frequent repetition rendered neceſſary—Whereas, if abſtinence from liquids

* Firſt Lines of the Practice of Phyſic, Vol. II. p. 353. 4th Edition.

† Ibid. Vol. I. p. 132.

be particularly attended to, one bleeding will have more effect, than three or four, if accompanied with that part of the antiphlogiftic regimen, and the lofs of blood be thereby prevented; which, confidering its importance in the conftitution, and the difficulty with which its lofs is made up, fhould be at all times avoided when poffible.

Of all cafes of hæmorrhagy, that from the lungs is the moft dangerous in its nature, and moft difficult of cure. This will appear evident if we recollect their particular ftucture, their large and numerous veffels, their conftant motion, &c.

As to their ftructure, anatomy demonftrates that they are compofed of a congeries of blood veffels, abforbents, and nerves, together with the air cells; and that all thefe are only connected by the cellular membrane, the common connecting medium of the body: for I do not mention their pleuritic covering, as I am only fpeaking of their fubftance. The blood veffels, with which alone our prefent fubject is connected, are very large, and in greater number than in any other part of the body of the fame fize. This was abfolutely neceffary to circulate the very large quantity of blood generally fent to them. Haller obferves *, that the quantity of

* Prim. Lin. Phyfiol. § 246.

blood which enters into the lungs is equal to, or even perhaps greater than, that which is ſent in the ſame time throughout the reſt of the body. And, as the chief buſineſs of the lungs is for reſpiration, by which they are kept conſtantly in action, ſo it will appear evident why hæmorrhages here are more dangerous, as well as more obſtinate, than in any other part, as their conſtant motion counteracts and prevents the union of the ruptured veſſel.

*Queen Anne Street, Eaſt,*

August 7, 1792.

